# Principal Component Regression Analysis of the Relation Between CIELAB Color and Monomeric Anthocyanins in Young Cabernet Sauvignon Wines

**DOI:** 10.3390/molecules13112859

**Published:** 2008-11-17

**Authors:** Fu-Liang Han, Wen-Na Zhang, Qiu-Hong Pan, Cheng-Rong Zheng, Hai-Yan Chen, Chang-Qing Duan

**Affiliations:** No. 17, Qinghua East Road, Haidian District, Center for Viticulture and Enology, College of Food Science & Nutritional Engineering, China Agricultural University, Beijing, 100083, P. R. China; E-mails: hfl109@yahoo.com.cn (F-L. H.), eventzh@yahoo.com.cn (W-N. Z.), panqiuhong2007@vip.sohu.com (Q-H P), 10400123@tinghsin.com.cn (C-R. Z.), hychenok@gmail.com (H-Y. C.)

**Keywords:** Anthocyanins, CIELAB color values, Wine color, Principal component regression.

## Abstract

Color is one of the key characteristics used to evaluate the sensory quality of red wine, and anthocyanins are the main contributors to color. Monomeric anthocyanins and CIELAB color values were investigated by HPLC-MS and spectrophotometry during fermentation of Cabernet Sauvignon red wine, and principal component regression (PCR), a statistical tool, was used to establish a linkage between the detected anthocyanins and wine coloring. The results showed that 14 monomeric anthocyanins could be identified in wine samples, and all of these anthocyanins were negatively correlated with the L*, b* and H*ab values, but positively correlated with a* and C*ab values. On an equal concentration basis for each detected anthocyanin, cyanidin-3-*O*-glucoside (Cy3-glu) had the most influence on CIELAB color value, while malvidin 3-*O*-glucoside (Mv3-glu) had the least. The color values of various monomeric anthocyanins were influenced by their structures, substituents on the B-ring, acyl groups on the glucoside and the molecular steric structure. This work develops a statistical method for evaluating correlation between wine color and monomeric anthocyanins, and also provides a basis for elucidating the effect of intramolecular copigmentation on wine coloring.

## Introduction

**Figure 1:**
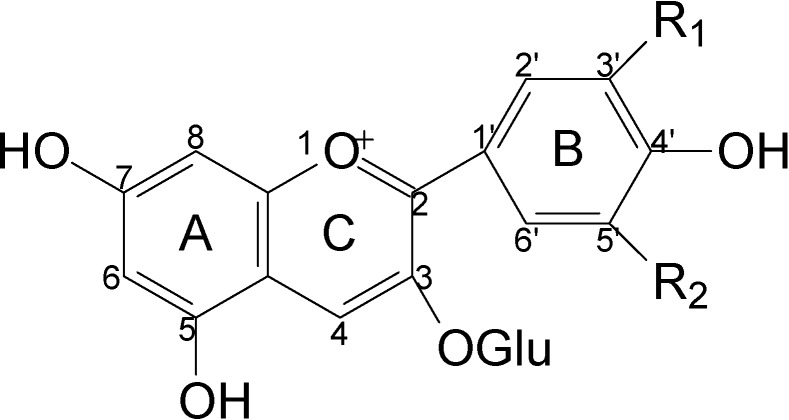
Structures of the five common anthocyanin monoglucosides.

Anthocyanins are the main pigments contributing to red wine color, and play an important role in wines’ organoleptic qualities. At present, there are several hundred known anthocyanins and anthocyanin-derived pigments with diverse structures and properties that have been detected in different wines or model wines. According to their structures, anthocyanins can be classified into the following groups: non-acylated anthocyanins (common anthocyanin monoglucosides in red wine), acylated anthocyanins, pyranoanthocyanins, direct flavanol-anthocyanin condensation products, acetaldehyde-mediated or other compound-mediated flavanol-anthocyanin condensation products, and polymeric anthocyanins [1,2,3,4,5,6]. In red wines made from *Vitis vinifera* L. grapes there are five common types of anthocyanin monoglucosides: the glucosides of delphinidin, cyanidin, petunidin, peonidin and malvidin (Figure 1). All other types are derived from them during grape berry development or wine fermentation and ageing [1,2,4,7].

Delphinidin 3-*O*-glucoside (Dp3-glu) R_1_-OH, R_2_-OH; Cyanidin 3-*O*-glucoside (Cy3-glu) R_1_-OH, R_2_-H; Petundin 3-*O*-glucoside (Pt3-glu) R_1_-OCH_3_, R_2_-OH; Peonidin 3-*O*-glucoside (Pn3-glu) R_1_-OCH_3_, R_2_-H; Malvidin 3-*O*-glucoside (Mv3-glu) R_1_-OCH_3_, R_2_-OCH_3_.

Red wine is a complex solution and wine coloring is affected by many factors such as concentrations and types of anthocyanins, polymeric pigments, wine pH, SO_2_ concentration, alcohol, metal ions, copigmentation, etc. [6,8,9,10,11,12,13,14,15]. Among these factors, the proportion of the contribution of intermolecular copigmentation is estimated to be up to 30%-50% in young red wine coloring, while polymerized pigments, are found to be the main contributors to color of aged red wine because of their stability towards pH and SO_2_ bleaching, which account for 35%-63% of wine color.. Therefore, in the constitution of young red wine color, anthocyanins themselves may account for 50%-70% since intermolecular copigmentation takes up 30-50%, which indicates that some intrinsic factors like intramolecular copigmentation may make great contribution to wine coloring [10]. It is generally believed that substituent species and numbers in structures of anthocyanins will affect, to some extent, wine color, which is called intramelocular copigmentation. However, it remains uncertain to what extent the various substituents determine wine coloring.

Since anthocyanins play a crucial role in wine coloring, it is essential to evaluate the relation between anthocyanins and wine color. The previous reports concerning influence of anthocyanins on wine color mainly involve pelargonidin, pyranoanthocysnins and five common anthocyanins including delphinidin 3-*O*-glucoside (Dp3-glu), cyanidin 3-*O*-glucoside (Cy3-glu), petunidin 3-*O*-glucoside (Pt3-glu), peonidin 3-*O*-glucoside (Pn3-glu) and malvidin 3-*O*-glucoside (Mv3-glu) (see Figure 1) [16,17,18,19,20]. Some statistical tools, such as unitary linear regression, Pearson’s correlation, principal component analysis and forward stepwise multiple linear regression, were used for evaluating relationship between the color and the anthocyanin compositions of red wines [21,22,23,24,25].

However, to our knowledge, another statistical tool, principal component regression (PCR), has still not been applied to describing the relationship between anthocyanins and color. In the process of evaluating the relation between anthocyanins and wine color by statistical tools, the problem often encountered is that multicollinearity, or high correlation among the independent variables in a regression equation, which makes the regression coefficient incapable of correctly identifying the contributors to the wine color. PCR combines linear regression and principal component analysis. It establishes a relationship between the dependent variable and the selected principal components of the independent variables, which allows the transformation of a set of correlated x-variables into an equal number of uncorrelated variables. As a result, analysis of PCR may help to solve the problem that occurs in wine color evaluation. The present study aims at developing a linkage between CIELAB color and monomeric anthocyanins during Cabernet Sauvignon wine fermentation, by means of PCR. Through this linkage, we tried to estimate contribution to wine coloring of various substituents in the anthocyanin structures.

## Results and Discussion

### Identification of monomeric anthocyanins in Cabernet Sauvignon wine

Fourteen anthocyanins were identified from Cabernet Sauvignon wine according to retention time, elution order, and MS/MS fragmentation characteristics of their molecular ions. These anthocyanins included five non-acylated anthocyanin monoglucosides and nine acylated anthocyanins, as listed in Table 1.

### Validity of Principal Component Regression (PCR)

Statistical analysis was carried out according to the process of principal component regression (PCR). It establishes a relationship between the dependent variable (Y, color parameters) and the selected principal components (Z_j_, j=1 … m, m≤p) of the independent variables (x_i_, i=1 … p, anthocyanins). These new variables (Z_j_) are all linear combinations of the original correlated x-variables.

**Table 1:** Monomeric anthocyanins in Cabernet Sauvignon wines.

Peak No.	Rt	Anthocyanins	λ_max_ (nm)	Molecular and Product Ions (m/z)
1	8.047	Dp3-glu	524	465 (M^+^), 303
2	10.272	Cy3-glu	516	449 (M^+^), 287
3	11.303	Pt3-glu	524	479 (M^+^), 317
4	13.272	Pn3-glu	518	463 (M^+^), 301
5	13.885	Mv3-glu	528	493 (M^+^), 331
6	14.612	Dp3-acet-glu	526	507 (M^+^), 303
7	17.490	Pt3-acet-glu	522	521 (M^+^), 317
8	19.814	Pn3-acet-glu	522	505 (M^+^), 301
9	20.212	Mv3-acet-glu	528	535 (M^+^), 331
10	21.367	Mv3-caff-glu	532	655 (M^+^), 331
11	21.793	Pt3-coum-glu	530	625 (M^+^), 317
12	22.836	Mv3-cis-coum-glu	536	639 (M+), 331
13	23.625	Pn3-coum-glu	522	609 (M^+^), 301
14	23.849	Mv3-trans-coum-glu	530	639 (M^+^), 331

Dp: Delphinidin; Cy: Cyanidin; Pt: Petunidin; Pn: Peonidin Mv: Malvidin; glu: glucoside; acet: acetyl; coum: coumaryl; caff: caffeoyl; acetal: acetaldehyde; pa: pyruvic acid

The number of Z_j_ selected was determined by examining the proportion of total variance explained by each component and the physical meaning of all partial regression coefficients. The selected Z_j_ were utilized as uncorrelated variables in the PCR model. Parameters were estimated by the following equation:

Y=*b_0_* + *b*_1_ * Z_1 _ + *b*_2_ * Z_2_ … *b*_m_ * Z_m_ + *e*

where *b_0_* is the intercept term, *b* is the regression coefficient, *Z* is the principal component *j*, *m* is the number of Z_j_ included in the regression, and *e* is the residual (error) term. These estimates of the regression coefficients were used to reconstitute regression coefficients for the original variables. As a result, the partial regression coefficients of equation obtained by PCR can compare the importance of variables, i.e. the contribution of anthocyanins to color. Some related theories of PCR can be found elsewhere [26,27,28,29].

The concentrations of 14 anthocyanins and CIELAB color values in all wine samples, respectively, were detected by using high performance liquid chromatography (HPLC) and by spectrophotometry (data not shown). PCR was used for analyzing correlation between the detected anthocyanins and the various CIELAB color values. The results showed that first principal component represented more than 80% of the total variance of independent variables in all wine samples. When only first principal component was considered, various anthocyanins were negatively correlated with the L*, b* and H*ab values, and were positively correlated with a* and C*ab values. However, when two or more principal components were selected, the correlation mentioned above disappeared, perhaps because the model was over-fitted. Therefore, only first principal component was selected in the model.

Table 2 showed the determination coefficients (R^2^) of the regression equations from the content individual anthocyanin versus the corresponding color parameter. Except for correlation with b* value in W3, W4 and W5 samples, the determination coefficients of the regression equations showed significant correlation in all other samples. Such an unapparent correlation with b* value in W3, W4 and W5 might be related to the addition of commercial pectolytic enzymes during winemaking, which led to more non-anthocyanin phenolic compounds present in these wines and significantly affected the b* value. 

**Table 2:** Determination coefficients (R^2^) of regression equations from anthocyanins vs. CIELAB values estimated by PCR in five wine samples.

Samples	L*	a*	b*	C*ab	H*ab
W1	0.6490**	0.7796**	0.1997**	0.7649**	0.5306**
W2	0.7623**	0.7347**	0.3134**	0.7309**	0.6013**
W3	0.7458**	0.7885**	0.0636	0.7846**	0.4416**
W4	0.5937**	0.6456**	0.0073	0.6405**	0.2111**
W5	0.7529**	0.8157**	0.0366	0.8197**	0.5087**
Significance	a	a	c	a	b

**: Significance at P < 0.05. Different letters indicate significant difference between these samples at P < 0.05

The ANOVA analysis showed that no significant difference among the detected samples was present in the determination coefficients of anthocyanins vs. L*, a* or C*ab values, but the determination coefficients of anthocyanins vs. b* or H*ab values were significantly different among these wine samples. It is conceived that anthocyanins made a greater contribution to L*, a* and C*ab values than to H*ab and b* values (Table 2). 

The anthocyanins in this study accounted for 64.56-81.57% of the a* value in wine color. The data was close to the report of Boulton, in which anthocyanins accounted for 50-70% of the color values in young red wines [10]. The result indicated that the intermolecular copigmentation, other non-detected anthocyanins and other factors totally accounted for the rest color of a* value (18.43-35.44%). Similarly, these 14 anthocyanins accounted for 59.37-76.23% of L* value, 0.73-31.34% of b* value, 64.05-81.97% of C*ab, 21.11-60.13% of H*ab value.

### The CIELa*b*color of different anthocyanins

Various anthocyanins had different partial regression coefficients (Table 3), representing different color values, which indicated that even a slight change in concentration of one anthocyanin with a bigger partial regression coefficient (absolute values for L*, b*, H*ab) would lead to remarkable change of one color parameter, on the basis of the same concentration of various anthocyanins. As can be seen from Table 3, Cy3-glu presented the largest color values at per unit content although compared with Mv3-glu, its content was very low, followed by Pt3-coum-glu and Pn3-coum-glu, while Mv3-glu made the least color values although it was the most abundant in young red wine. These results indicated that if the concentrations of them were same, the anthocyanins making the larger color values will contribute more to color present.

### Effects of substituents of the B-ring on anthocyanins’ contribution to color

According to parameters shown in Table 3, the color L*, b* and H*ab values of five common anthocyanin monoglucosides increased and the color a* and C*ab values decreased as the number of substituents on the B-ring grew. That is, Cy3-glu and Pn3-glu (two substituents) had greater color values than did Dp3-glu, Pt3-glu and Mv3-glu (three substituents). Simultaneously, the color L*, b* and H*ab values of these five anthocyanins increased, while the color a* and C*ab values of them decreased as the number of methoxyl groups grew. That is, the order of the five anthocyanins monoglucoside’s color values was Dp3-glu (three hydroxyls, no methoxyl) > Pt3-glu (two hydroxyls, one methoxyl) > Mv3-glu (one hydroxyl, two methoxyls) (only in order, same as below). Therefore, it can be concluded that hydroxyl groups contributed more to both the decrease of L*, b* and H*ab parameters and the increase of a* and C*ab ones, compared with methoxyl groups. This result was consistent with what was reported by Heredia *et al.*, in which it was concluded that the absorbance decreased when hydroxyl groups were substituted by methoxyl groups, according to comparison of three (Dp3-glu > Pt3-glu > Mv3-glu) and two (Cy3-glu > Pn3-glu) B-ring substituted anthocyanins at pH 1.5 and pH 3.5 [16]. In addition, Cabrita *et al.* also pointed out that Cy3-glu had the maximum absorbance among five anthocyanin monoglucosides [17]. 

By comparing the effects of various substituents at C5’ position on wine color (Table 3), it could be found that the color parameters of the five common anthocyanin monoglucosides (absolute values for color L*, b* and H*ab parameters) followed this order: Cy3-glu (3’,4’-OH, 5’-H) > Dp3-glu (3’,4’-OH, 5’-OH); Pn3-glu (3’-OCH3, 4’-OH, 5’-H) > Pt3-glu (3’-OCH3, 4’-OH, 5’-OH) > Mv3-glu (3’-OCH3, 4’-OH, 5’-OCH3). Thus, it can be concluded that the substituent of C5’ on the B-ring decreased the a* and C* values and increased the L*, b* and H*ab values, i.e., it weakened the contribution of anthocyanins to color, and the methoxyl group addition weakened the contribution to color more than hydroxyl group addition did. 

As a result, it is suggested that to a large extent, the color of the five common anthocyanin monoglucosides depended on the number, position and type of substituents on B-ring. In addition, the form and proportion of anthocyanins in wine also affected wine coloring. At wine pH, each form of anthocyanins comprises a proportion of the total at equilibrium. In this way, the colorless forms of Mv3-glu may have comprised a higher proportion so that this pigment endows the least color value [16,30].

### Effect of acylation of anthocyanins on color

In contribution proportion to wine coloring, the acetylated forms of the common anthocyanin monoglucosides differed from their parent anthocyanin monoglucosides. The color values of acetylated anthocyanins showed, in sequence, Pt3-acet-glu > Pn3-acet-glu > Dp3-acet-glu > Mv3-acet-glu, while for their parent anthocyanin monoglucosides, the color values were Cy3-glu > Pn3-glu > Dp3-glu > Pt3-glu > Mv3-glu (Table 3).

The results indicated that the addition of acetyl group would affect the color of anthocyanins monoglucosides. However, almost no significant difference was found in analysis of ANOVA concerning correlation between color values of acetylated anthocyanins and its corresponding parent anthocyanins, suggesting that the impact of acetyl group may be limited, because of less intramolecular copigmentation on the chromophore [31].

**Table 3:** The color values of various anthocyanins estimated by PCR.

Peak No.	Anthocyanins	L*		a*		b*			C*ab			H*ab		
		Mean ± STD		Mean ± STD			Mean ± STD			Mean ± STD			Mean ± STD	
2	Cy3-glu	-2.3079±0.3995	a	2.8100±0.5751	a		-0.2977±0.3165	a		2.6948±0.4598	a		-1.3099±1.0863	a
11	Pt3-coum-glu	-1.7715±0.3522	b	2.1636±0.5072	b		-0.1840±0.1485	ab		2.0912±0.5269	b		-0.8846±0.4949	ab
13	Pn3-coum-glu	-1.2023±0.1639	c	1.4641±0.2424	c		-0.1273±0.1024	bc		1.4149±0.2672	c		-0.5971±0.3205	bc
12	Mv3-cis-coum-glu	-1.1452±0.196	c	1.3910±0.2568	c		-0.1175±0.0844	bc		1.3456±0.2818	c		-0.5578±0.2548	bcd
10	Mv3-caff-glu	-0.8647±0.0532	d	1.0521±0.1110	d		-0.0972±0.0853	bc		1.0146±0.1225	d		-0.4449±0.2722	bcde
7	Pt3-acet-glu	-0.3247±0.0242	e	0.3945±0.0401	e		-0.0376±0.0333	c		0.3799±0.0389	e		-0.1715±0.1076	cde
4	Pn3-glu	-0.2644±0.0272	e	0.3208±0.0344	ef		-0.0323±0.0307	c		0.3083±0.0250	ef		-0.1435±0.1001	cde
8	Pn3-acet-glu	-0.2180±0.0255	ef	0.2644±0.0308	ef		-0.0239±0.0190	c		0.2553±0.0353	ef		-0.1104±0.0587	cde
1	Dp3-glu	-0.1786±0.0212	ef	0.2172±0.0325	ef		-0.0218±0.0211	c		0.2087±0.0269	ef		-0.0981±0.0708	cde
6	Dp3-acet-glu	-0.1760±0.0123	ef	0.2133±0.0129	ef		-0.0200±0.0170	c		0.2056±0.0155	ef		-0.0911±0.0527	cde
14	Mv3-trans-coum-glu	-0.1179±0.0047	ef	0.1432±0.0101	ef		-0.0139±0.0129	c		0.1378±0.0083	ef		-0.0624±0.0417	de
3	Pt3-glu	-0.1116±0.0085	ef	0.1356±0.0134	ef		-0.0127±0.0110	c		0.1306±0.0139	ef		-0.0583±0.0352	de
9	Mv3-acet-glu	-0.0196±0.0012	f	0.0237±0.0009	f		-0.0022±0.0019	c		0.0229±0.0015	f		-0.0101±0.0057	e
5	Mv3-glu	-0.0091±0.0007	f	0.0110±0.0005	f		-0.0010±0.0008	c		0.0106±0.0008	f		-0.0047±0.0026	e

Different letters indicate significant differences at P < 0.05 (n=5); these values were the partial regression coefficients of equations obtained by PCR analysis.

Coumarylated anthocyanins (Pt3-coum-glu, Pn3-coum-glu, Mv3-cis-coum-glu, Mv3-trans-coum-glu) decreased the L*, b* and H*ab values and increased the a* and C*ab values, compared with their corresponding acetylated anthocyanins (Pt3-acet-glu, Pn3-acet-glu, Mv3-acet-glu) and their original anthocyanins (Pt3-glu, Pn3-glu, Mv3-glu) although some of them were not significant to b* and H*ab values (Table 3). It was suggested that coumarylated anthocyanins made a greater contribution to color parameters than their corresponding acetylated anthocyanins and original anthocyanins did, because the aromatic ring of the coumaryl could stack with the chromophore of anthocyanins to result in a stronger intramolecular copigmentation [7,12,18,21,32,33,34,35,36]. The Mv3-cis-coum-glu presented a greater color than the Mv3-trans-coum-glu did, perhaps because the *cis*-coumaryl residue is almost parallel to the anthocyanidin moiety, while the *trans*-coumaryl residue presents a quasi-perpendicular conformation [35]. Thus, the estimated contributions of *cis*-coumaryls to the color values was greater than that of *trans*-coumaryl residues.

Caffeoyl addition in Mv3-glu decreased color L*, b* and H*ab values, and increased a* and C*ab values more than *trans*-coumaryl did, but less than *cis*-coumaryl did (Table 3). Caffeoyl residues (*cis*- and *trans*- isomers) may share a similar mechanism of intramolecular copigmentation with coumaryl residues (*cis*- and *trans*- isomers) [35]. Generally, the *trans*-form is stabler than the *cis*-form and it is the predominating structure *in vivo* [35]. Therefore, caffeoylated anthocyanin detected in the study should be the *trans*-caffeoylated anthocyanins. According to intramolecular copigmentation, *trans*-caffeoylated anthocyanins should present greater color value than their corresponding *trans*-coumarylated anthocyanins, because the former had one hydroxyl group, besides the coumaryl residues. According to intramolecular copigmentation of caffeoyl and coumaryl residues and their structures, the following assumptions can also be deduced: the *cis*-caffeoylated anthocyanins should make a greater contribution to wine coloring than their corresponding *cis*-coumarylated anthocyanins; the contribution of *cis*-caffeoylated anthocyanins to color was greater than that of the corresponding *trans*-caffeoylated anthocyanins, which still needs to be confirmed.

## Conclusions

The regression equations of anthocyanins vs. color of L*, a*, C*ab, H*ab values were significant at level of 0.05. Various anthocyanins were negatively correlated with the L*, b* and H*ab values and were positively correlated with a* and C*ab values. Monomeric anthocyanins detected in this study accounted for 64.56-81.57% of a* value, 59.37-76.23% of L* value, 0.73-31.34% of b* value, 64.05-81.97% of C*ab, 21.11-60.13% of H*ab value. These results indicated that principal component regression was a suitable method for evaluating the relation between anthocyanins and their color values, especially the color L*, a* and C*ab values.

Among the detected anthocyanins, Cy3-glu showed the highest color value, followed by Pt3-coum-glu, whereas Mv3-glu had the least color value. These results indicated that the color values of various anthocyanins were influenced by their structures, substituents on the B-ring, acyls on the glucoside, and the molecular steric structure. This conclusion will be helpful for understanding the coloring mechanism of anthocyanins and dynamics of red wine color. 

## Materials and methods

### Analytical standards and reagents

Acetonitrile (HPLC grade) and formic acid (96%) were purchased from the Fisher Company (Fairlawn, NJ, USA). Malvidin-3-glucoside chloride was purchased from Extrasynthese SA (Genay, France).

### Samples

Grape berries (*V. vinifera* cv Cabernet Sauvignon) were processed in the 2005 vintage. For all five wine samples (W1, W2, W3, W4, and W5), the maceration and fermentation were carried out in stainless steel tanks (500 L). To each must SO_2_ (50 mg/L) was added before alcohol fermentation, then, the yeast (*Saccharomyces cerevisiae* VR5, Netherlands, 0.2 g/L) was added in the next day. Wine samples W3, W4 and W5 had 25 mg/L of commercial pectolytic enzymes FCE, Ex-color, and Tinto added (Lallemand Inc, France), respectively, while wine samples W1 and W2 did not, and the grape berries for sample W2 were treated with Azomite fertilizer™ (USA). The temperature of alcohol fermentation ranged from 16–26 ºC. After alcohol fermentation finished, maceration and malolactic fermentation (MBR B1, Lallemand S.A., France, 0.02 g/L, temperature kept about 18 ºC) took place, and SO_2_ (50 mg/L) was added at the end of malolactic fermentation. Wine samples were collected from the tanks every other day during alcohol fermentation as well as at the beginning and at the end of malolactic fermentation, respectively. All the experiments were carried out in replicate, and each sample was independently analyzed twice.

### Analysis of CIELAB values

Analysis of CIELAB values were performed according to the method described by Ayala *et al.* [37,38]. Coordinate a* is related to red color if a*>0 and to green color if a*<0; Analogously, coordinate b* is related to yellow color if b*>0 and to blue color if b*<0; L* (lightness) is the lightness of a colored object judged relative to the lightness of that appears as white; C*ab (chroma) is the chromaticness of a colored object judged relative to the white; H*ab (hue) is the attribute of appearance by which a color is identified according to its resemblance to red, yellow, green, or blue, or a combination of two of these in sequence.

All the wine samples were filtered through 0.45 µm filters (cellulose acetate and nitrocellulose, CAN) prior to direct analysis without dilution. Spectrophotometric measurements were carried out with a Shimadzu UV-2450 spectrophotometer, 0.2 cm path length, at 440 nm, 530 nm, and 600 nm, respectively. Distilled water was used as the blank. The color parameters (L*, a*, b*, C*ab, H*ab) were calculated from CIELAB, thus, the tri-dimensional color space was obtained. All analyses were replicated twice.

### Quantitative analysis of anathocyanins by HPLC-MS

All the wine samples were filtered through 0.45 µm filters (cellulose acetate and nitrocellulose, CAN) and the resulting filtrates were directly used for quantitative analysis without dilution. An Agilent 1100 series LC-MSD trap VL, equipped with a DAD detector and reversed phase column (Kromasil C18 250 × 4.6 5μm), was used. The solvents were: (A) aqueous 2% formic acid, and (B) acetonitrile containing 2% formic acid. The gradient was from 6% to 10% B for 4 min, from 10% to 25% B for 8 min, isocratic 25% B for 1 min, from 25% to 40% for 7 min, from 40% to 60% for 15 min, from 60% to 100% for 5 min, from 100% to 6% for 5 min, at a flow rate of 1.0 mL/min. Injection volumes were 30 μL, and the detection wavelength was 525 nm. MS conditions were as follows: Electrospray ionisation (ESI) interface, positive ion model, 35 psi nebulizer pressure, 10 mL/min dry gas flow rate, 350 ºC dry gas temperature, and scans at *m/z* 100–1000. All analyses were replicated twice.

### Statistical analysis

All individual anthocyanins were quantified and expressed as malvidin 3-glucoside content from the chromatographic results. If any of these anthocyanins was not detected in a sample, they were represented by zero in the data matrix which was conducted standardization prior to PCR analysis. PCR was performed with the statistical software of SAS V8 (SAS Institute, USA). Analysis of variance (ANOVA) was assessed with DPS 3.01 (DPS data processing system, Qiyi Tang, China), and significant differences (*P* < 0.05) were identified using the Duncan’s multiple range tests.
